# Metabolic insufficiency underlies intratumoral cytotoxic T cell dysfunction

**DOI:** 10.1186/2051-1426-3-S2-P399

**Published:** 2015-11-04

**Authors:** Tushar Gupta, Nicole E Scharping, Rebecca S Moreci, Greg M Delgoffe

**Affiliations:** 1 University of Pittsburgh, Pittsburgh, PA, USA

## 

T cells have the remarkable ability to recognize and remove abnormal cells with precision, a feature that is very desirable for the treatment of cancer. However, while T cells specific for tumor antigens are primed and can infiltrate tumors, they are quickly rendered dysfunctional, through both cell intrinsic and cell extrinsic mechanisms. One way that tumors cripple T cell function is through the generation of an immunosuppressive microenvironment that is chronically inflamed, hypoxic, and nutrient poor. T cell activation and subsequent generation of effector function is bioenergetically demanding, requiring large amounts of metabolic intermediates to effectively proliferate, produce cytokines, and lyse target cells. We hypothesized that T cell dysfunction in cancer is due, in part, to metabolic insufficiency caused by chronic activation in metabolically dearth conditions. Using single-cell metabolic assays and extracellular flux analysis, we show that CD8^+^ cytotoxic T cells that infiltrate tumors demonstrate a progressive loss of mitochondrial function and mass, concomitant with upregulation of markers that correlate with T cell exhaustion. This mitochondrial dysfunction occurs independently of coinhibitory molecule signaling and specifically in the tumor microenvironment. This results in a failure to generate an adequate pool of ATP and in inability to effectively translate effector gene transcripts. This in stark contrast to T cells responding to an acute viral infection, where activated effector T cells demonstrate increased mitochondrial mass and ATP reserve. Further, artificial induction of mitochondrial dysfunction in T cells results in upregulation of coinhibitory molecules and an ‘exhausted-like’ phenotype, suggesting that metabolic insufficiency underlies the dysfunctional phenotype in cancer. Taken together, our data support a model in which tumor-infiltrating T cells have metabolic needs that cannot be met, resulting in failed effector function and tumor growth. Our studies also suggest that modulation or reprogramming of the altered metabolism of intratumoral T cells represents a potential strategy to reinvigorate dysfunctional T cells for the immunotherapeutic treatment of cancer.

